# Complete Genome Sequence of a Serotype A Foot-and-Mouth Disease Virus from an Outbreak in Saudi Arabia during 2015

**DOI:** 10.1128/genomeA.01591-15

**Published:** 2016-01-21

**Authors:** K. Bachanek-Bankowska, J. Wadsworth, B. Thapa, D. P. King, N. J. Knowles

**Affiliations:** The Pirbright Institute, Pirbright, Woking, Surrey, United Kingdom

## Abstract

The complete genome of a foot-and-mouth disease (FMD) type A virus isolated from cattle in Saudi Arabia in 2015 is described here. This virus belongs to an FMD virus lineage named genotype VII, which is normally endemic on the Indian subcontinent.

## GENOME ANNOUNCEMENT

Foot-and-mouth disease (FMD) virus is a single-stranded positive-sense RNA of the genus *Aphthovirus* (family *Picornaviridae*), which causes an economically important vesicular disease. FMD spreads rapidly among cloven-hooved animals, leading to a loss of or reduction in livestock production, which additionally disrupts international trade. Seven immunologically distinct serotypes of the virus exist: O, A, C, Southern African Territories (SAT) 1, SAT 2, SAT 3, and Asia 1, each containing numerous variants often restricted to specific geographical locations (topotypes) (1). The nonenveloped virus particle contains a genome of approximately 8.3 kb. Due to a high mutation rate, new FMD virus lineages can emerge unpredictably to pose challenges to local and international disease control strategies.

During September 2015, the FAO World Reference Laboratory for FMD (WRLFMD), located at the Pirbright Institute, United Kingdom, received samples from cattle displaying clinical signs of FMD in Saudi Arabia. These animals were previously vaccinated against three serotypes of FMD virus (FMDV) (O, A, and SAT 2) with a high-potency vaccine. Phylogenetic analysis of the VP1 coding sequence of the virus isolates from three farms revealed a close relationship to FMD type A viruses from the Indian subcontinent. These were unexpected findings, since FMDV outbreaks due to serotype A have not been reported in Saudi Arabia since 2005, when a virus belonging to the A-Iran-05 lineage (which is widespread in western Asia) was detected (2).

Here, we report the complete genome sequence of the A/SAU/1/2015 strain from the A/ASIA/G-VII lineage causing outbreaks in Saudi Arabia since September 2015. The virus was isolated from a clinical sample by a single passage in primary bovine thyroid (BTy) cells. The genome was sequenced using MiSeq technology (Illumina, USA), as previously described (3). Sequence analyses were undertaken using SeqMan NGen and SeqMan Pro version 12 (Lasergene package; DNAStar, Inc., Madison, WI).

The genome was 8,192 nucleotides (nt) in length and included 18 nt of a poly(C) tract of unknown length located within the 5′ untranslated region. A single large open reading frame of 6,999 nt was predicted to encode a putative polyprotein of 2,333 amino acids (aa) containing four structural (VP4, VP2, VP3, and VP1) and 10 nonstructural (L, 2A, 2B, 2C, 3A, 3B1, 3B2, 3B3, 3C, and 3D) proteins. The most closely related publicly available complete genome sequence to A/SAU/1/2015 was isolated in 2013 from Gazipur, Bangladesh (that of BAN/GA/Sa-197/2013, GenBank accession no. HM854025) (4), with which it shared 96.3% nucleotide identity. Both sequences contain a deletion at an antigenically significant residue within VP3 (VP3^59^). This deletion was previously reported in other closely related isolates circulating in India (5).

Viruses belonging to the A/ASIA/G-VII lineage were initially identified in the Indian state of Assam in November 2002, from where they rapidly spread to five other Indian states, including Gujarat and Karnataka, by February 2003 (6). The recent appearance and spread of the A/ASIA/G-VII lineage outside the Indian subcontinent reinforces the need to monitor the emergence of new viruses and understand the global epidemiology of FMDV in order to develop appropriate diagnostic and vaccine strategies.

### Nucleotide sequence accession number. 

The nucleotide sequence of FMDV A/SAU/1/2015 has been deposited in GenBank under accession number KU127247.
